# Supplementary material for: Relationship between Nitrogen Dynamics and Key Microbial Nitrogen-Cycling Genes in an Intensive Freshwater Aquaculture Pond
Source: Microorganisms. 2024 Jan 26;12(2):266. doi: 10.3390/microorganisms12020266 (PMC10892730; doi:10.3390/microorganisms12020266)
Supplement: Supplementary file 1 [file microorganisms-12-00266-s001.zip › microorganisms-2836899-supplementary.pdf]

Table S1. The Monte Carlo permutation test within the redundancy analysis of the relationships between environmental variables and gene abundances in the water column

Name	Explains %	Contribution %	pseudo-F	P
SPM flux	21.2	28.6	14	0.002
TN	11.9	16	9	0.002
SD	9.5	12.8	8.3	0.002
NO_3^--N	4.7	6.4	4.4	0.008
T	4	5.5	4	0.004
TP	2.7	3.6	2.7	0.024
NO_2^--N	2.8	3.8	3	0.028
NH_4^+-N	3.3	4.5	3.8	0.014
DO	2.1	2.8	2.5	0.058
Chl-a	2.9	3.9	3.5	0.012
PO_4^-	1.9	2.5	2.4	0.066
TN_{SPM} flux	2.8	3.7	3.7	0.018
TP_{SPM} flux	2.3	3.1	3.3	0.028
pH	2	2.7	3	0.038

Table S2. The Monte Carlo permutation test within the redundancy analysis of the relationships between environmental variables and gene abundances in the sediment

Name	Explains %	Contribution %	pseudo-F	P
NH_4^+-N flux	20.8	27.8	13.7	0.002
NO_3^--N flux	12	16	9.1	0.002
TN_{SPM} flux	9.3	12.4	8	0.004
O-NO_3^--N	10.3	13.8	10.6	0.002
TN_S	3.6	4.8	4	0.004
NO_2^--N flux	3.4	4.6	4	0.01
TP_{SPM} flux	3.9	5.2	4.9	0.004
O-NO_2^--N	2.8	3.8	3.8	0.022
TP_S	2.8	3.7	3.9	0.014
O-NH_4^+-N	2	2.6	2.9	0.022
O-pH	1.9	2.5	2.9	0.038
SPM flux	1	1.3	1.5	0.168
O-T	0.6	0.8	0.9	0.428
O-DO	0.6	0.8	0.9	0.408
